# Plasma osteopontin as a marker for future organ damage in pediatric systemic lupus erythematosus

**DOI:** 10.1186/1546-0096-10-S1-A123

**Published:** 2012-07-13

**Authors:** Ornella J Rullo, Jennifer MP Woo, Alice DC Hoftman, Miriam F Parsa, Gil Amarilyo, Deborah K McCurdy, Betty P Tsao

**Affiliations:** 1 UCLA, Manhattan Beach, CA, USA; 2 UCLA, Los Angeles, CA, USA; 3 UCLA School of Medicine, Los Angeles, CA, USA

## Purpose

Osteopontin (OPN) is a secreted phosphoprotein functioning in monocyte and T cell adhesion and migration. Expression of OPN in alternatively-activated macrophages has been implicated in post-inflammatory fibrosis of lung and kidney. In our previously conducted cross-sectional study of 72 pediatric SLE patients (pSLE; age of onset <18 years), increased circulating plasma OPN (cOPN) was observed in pSLE compared with healthy controls, and was associated of with ACR/SLICC damage index (SDI) scores >1 (p = 0.0007 and 0.001, respectively), although there was no association with SLEDAI. We therefore propose that OPN is a marker for pSLE disease progression, and that increased cOPN levels may predict the development of organ damage.

## Methods

17 pSLE patients have been followed for at least 6 months with cOPN measured by ELISA at baseline and at 6 month intervals. Serum Cr, urine protein/creatinine and GFR were recorded at baseline and every 6 months. SLEDAI was recorded at 3 month intervals, and at disease flare, and SDI at every 6 months. Cumulative disease activity (adjusted-mean SLEDAI, or AMS) was calculated by finding the area under the curve of serial SLEDAI measurements. Statistical analysis was performed using Student’s t test, Pearson’s correlation, and Fisher’s exact test.

## Results

6 of the 17 longitudinal subjects accumulated damage as per an increase in SDI during the course of the study (3 in the renal portion, 1 peripheral vascular, 1 pulmonary hypertension, 1 avascular necrosis). 5 of the 6 patients who accumulated damage had high cOPN levels (cOPN in the top quartile) in at least 1 of the previous 2 visits prior to the increase on SDI. 80% of subjects with a high cOPN had an increase in SDI, whereas only 11% of subjects with a cOPN in the bottom three quartiles had an increase in SDI, indicating an increase in SDI was more likely in those patients with a preceding high cOPN (p = 0.01). Cumulative disease activity (AMS) was associated with an increase in SDI scores (p < 0.0001) in this cohort, and was correlated with cOPN levels (Spearman r = 0.46, p = 0.02).

## Conclusion

cOPN is correlated with cumulative disease activity and may be a potential marker for future organ damage in pSLE. The rapid and/or early accrual of organ damage in patients with SLE has been associated with poorer outcomes, therefore a marker of risk for irreversible damage could be a critical component for guiding treatment decisions in pSLE.

## Disclosure

Ornella J. Rullo: None; Jennifer M.P. Woo: None; Alice D.C. Hoftman: None; Miriam F. Parsa: None; Gil Amarilyo: None; Deborah K. McCurdy: None; Betty P. Tsao: None.

